# A requirement for *Acinetobacter baumannii* purine biosynthesis during lung infection is exacerbated by host zinc deficiency

**DOI:** 10.1128/msphere.00735-25

**Published:** 2026-02-09

**Authors:** Lauren D. Palmer, Hannah R. Noel, Kacie A. Traina, John H. Geary, Eric P. Skaar

**Affiliations:** 1 Department of Microbiology and Immunology, University of Illinois Chicago 14681, Chicago, Illinois, USA; 2 Department of Pathology, Microbiology, and Immunology, Vanderbilt University Medical Center 12328, Nashville, Tennessee, USA; 3 Vanderbilt Institute for Infection, Immunology, and Inflammation, Vanderbilt University Medical Center 12328, Nashville, Tennessee, USA; University of Galway, Galway, Ireland

**Keywords:** *Acinetobacter baumannii*, zinc deficiency, pneumonia, multidrug resistant organisms, antimicrobial resistance, Tn-seq, purine biosynthesis

## Abstract

*Acinetobacter baumannii* is a leading cause of hospital-acquired infections, including ventilator-associated pneumonia. Dietary zinc deficiency is a major risk factor for pneumonia, and hospitalized patients at risk for *A. baumannii* infection have increased rates of zinc deficiency. We previously showed that dietary zinc deficiency enhanced *A. baumannii* pneumonia pathogenesis via an IL-13-dependent mechanism in mice. Here, we identified *A. baumannii* genes required for proliferation in the lungs of zinc-deficient mice using a genome-wide transposon sequencing (Tn-seq) screen. In zinc-deficient mice, Tn insertions in 614 *A. baumannii* genes led to significant differences in fitness in the lungs at 24 h. Most of these genes were also required in zinc-sufficient control mice. Mutants with disruptions in genes in the purine biosynthetic pathway, such as *purI*, and acinetobactin iron siderophore pathways were more strongly selected during lung infection in zinc-deficient mice compared to zinc-sufficient mice by Tn-seq. A reconstructed *purI* mutant was defective compared to wild type during lung infection in zinc-sufficient mice, with the defect further exacerbated in zinc-deficient mice. Thus, *A. baumannii* purine biosynthesis is required to infect the lung, and its requirement is exacerbated in a zinc-deficient host.

**IMPORTANCE:**

Dietary zinc deficiency is a major risk factor for infection worldwide. In the United States, hospitalized patients are at increased risk of zinc deficiency and *A. baumannii* pneumonia. In this study, *A. baumannii* purine biosynthesis was required for lung infection of mice, independent of dietary zinc. Therefore, bacterial purine biosynthesis is an attractive drug target for treating lung infections in patients with variable dietary zinc statuses, such as in hospitalized patients.

## OBSERVATION

*Acinetobacter baumannii* causes multidrug-resistant healthcare-associated infections, including ventilator-associated pneumonia (1, 2). Patients at increased risk for *A. baumannii* infection are also at increased risk of dietary zinc (Zn) deficiency, as we previously discussed (3). Zn deficiency compromises immune system function (4). We reported that dietary Zn deficiency promotes *A. baumannii* pathogenesis during lung infection through an IL-13-dependent mechanism (3). During infection, invading pathogens must acquire all nutrients in the host, while the host restricts nutrients in a process termed nutritional immunity (5). *A. baumannii* pathways important for infection in Zn-deficient mice are potential therapeutic targets for infections in high-risk patient populations. Here, we define the *A. baumannii* pathways required to proliferate in the lungs of Zn-deficient mice.

### Functional genomics identify *A. baumannii* pathways required for lung infection in Zn-deficient mice

**Fig 1:**
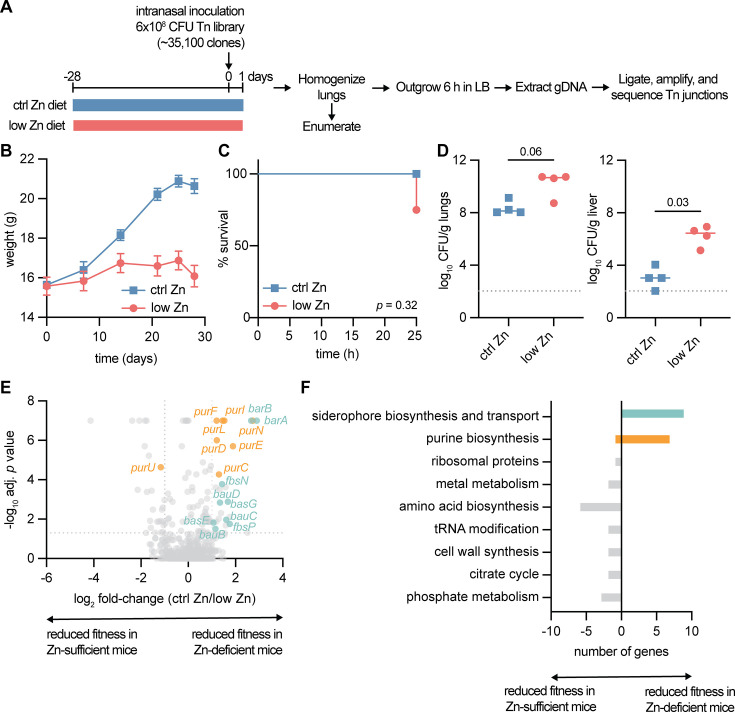
Identification of pathways *A. baumannii* uses to infect the lungs of Zn-deficient mice by Tn-seq. (**A**) Experimental design. (**B**) Mouse weight (*n* = 5, mean ± SEM). (**C**) Survival following intranasal infection with *A. baumannii* (*n* = 4; *P* by Mantel-Cox test). (**D**) Bacterial colony-forming units (CFU) enumerated from the lungs and livers. Each dot is from one mouse (*P* by Mann-Whitney test). Dotted line is limit of detection. (**E**) Differential selection against Tn mutants in the lungs of ctrl Zn v. low Zn diet-fed mice. Each dot is one gene. Dots for genes in siderophore biosynthesis and transport are colored teal and dots for genes in purine biosynthesis are colored orange. Dotted lines at |log_2_ fold-change| = 1 and *P* = 0.05. (**F**) Pathway analysis of genes differentially selected against in the lungs of Zn-sufficient mice (displayed as <0) or Zn-deficient mice (displayed as >0).

A transposon (Tn) library was generated from ~35,100 colonies of *A. baumannii* 17978UN with plasmid pJNW684 carrying a *himar1* derivative (6). The Tn library was inoculated into mice fed a low Zn diet (<0.5 mg Zn/kg chow; Zn-deficient) or control (ctrl) Zn diet (29 mg Zn/kg chow; Zn-sufficient) (Fig. 1A) (3). Prior to infection, mice fed the low Zn diet gained less weight than mice fed the ctrl Zn diet from 3.5 to 7.5 weeks of age (Fig. 1B). After infection, 4/4 Zn-sufficient mice and 3/4 Zn-deficient mice survived (Fig. 1C). The Zn-deficient mice had increased *A. baumannii* burdens in the lungs and dissemination to the livers at 24 h post infection (hpi) (Fig. 1D), as described (3).

The Tn library was outgrown from the lungs, and Tn junctions were amplified, sequenced, and mapped to the *A. baumannii* 17978UN genome (Table S1). Tn insertions in 624 and 614 genes reduced fitness of *A. baumannii* in the lungs of Zn-deficient and -sufficient mice, respectively (log_2_-fold-change ≤ −1 and adjusted *P* value ≤ 0.05). Of these, 553 genes were common to infection in the lungs of Zn-sufficient or Zn-deficient mice, and 122 were also among the 193 genes previously identified to be essential for persistence in mouse lungs by these cutoffs (Fig. S1) (6). Tn insertions in 26 genes led to significantly reduced fitness (adjusted *P* value ≤ 0.05) in Zn-sufficient v. Zn-deficient and 25 genes vice versa (Fig. 1E). Pathway analysis showed that many genes that were more important for persisting to 24 h in the lungs of Zn-deficient compared to Zn-sufficient mice encoded proteins involved in siderophore biosynthesis, transport of iron, or purine biosynthesis (Fig. 1F). These pathways were further interrogated to identify *A. baumannii* processes important for persistence in the lungs of Zn-deficient mice.

### Transport of the siderophore acinetobactin and purine biosynthesis are important for lung infection

**Fig 2:**
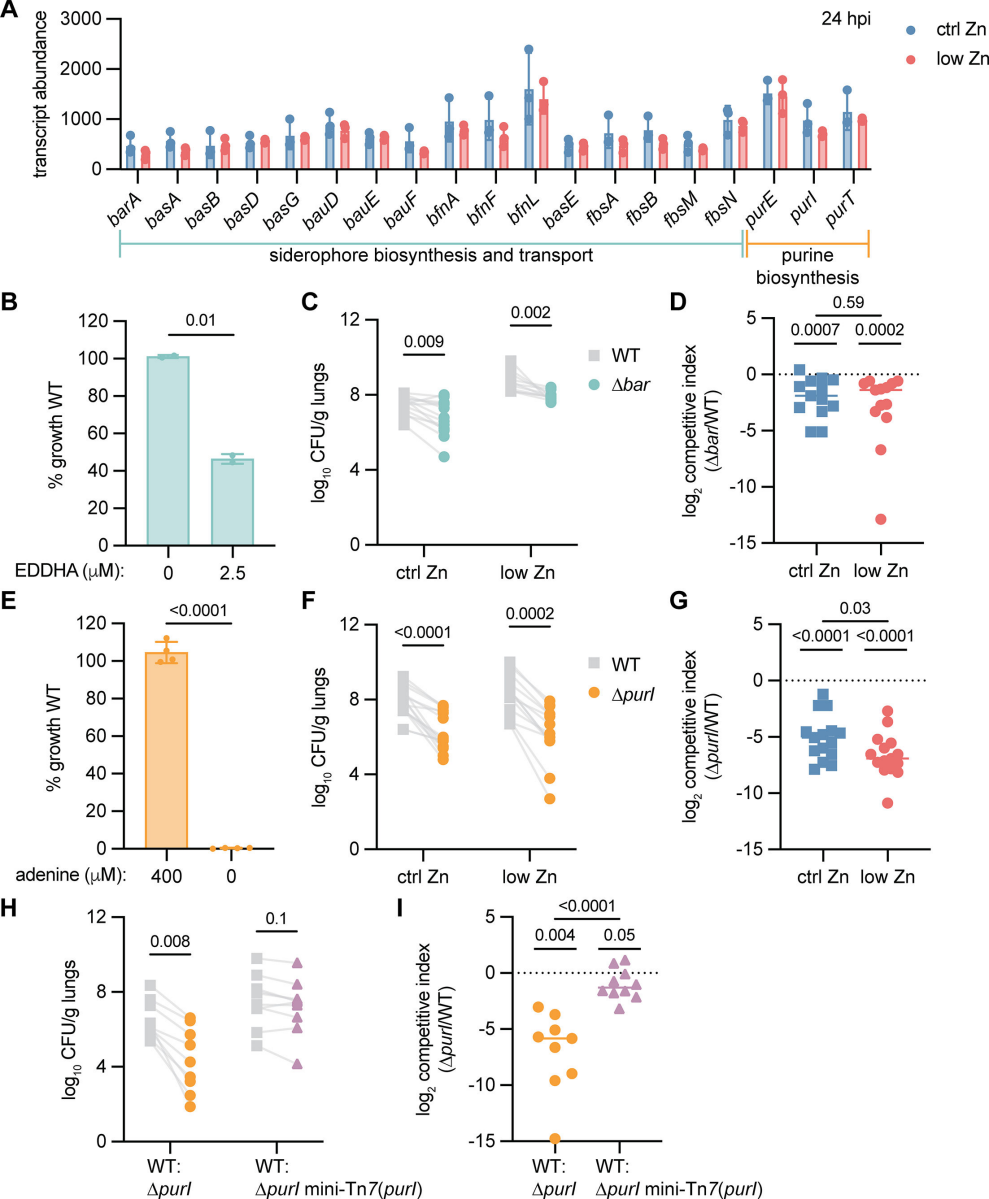
Purine biosynthesis is required to proliferate in the lungs of Zn-deficient mice. (**A**) Transcript abundance in the lungs of mice fed ctrl Zn or low Zn diet at 24 h post infection (hpi) measured by nanoString. Each dot is from one mouse (*n* = 3; mean ± SD; *P* ≥ 0.1 by multiple Mann-Whitney tests). (**B**) Percent growth of ∆*bar* compared to wild type (WT) in M9 pyruvate metal-free medium with and without iron limitation with ethylenediamine-N,N′-bis((2-hydroxyphenyl)acetic acid) (EDDHA) at 24 h. Each dot is one culture (*n* = 2; mean ± SD, *P* by Welch’s *T* test). (**C**) Bacterial colony-forming units (CFU) enumerated from the lungs of mice co-inoculated with WT and ∆*bar*. Lines connect CFU from a single mouse (ctrl zn, *n* = 13; low zn, *n* = 11; data combined from two experiments; *P* by multiple Wilcoxon tests with Bonferroni-Dunn post-test). (**D**) Competitive index of ∆*bar*/WT in the lungs of mice from (**C**). Each dot is from one mouse (median is shown; *P* by one-sample Wilcoxon test compared to 0 and comparison between diets by Mann-Whitney test). (**E**) Percent growth of ∆*purI* compared to wild type (WT) in the M9 pyruvate medium with and without adenine at 22 h. Each dot is one culture (mean ± SD, *P* by Welch’s *T* test). (**F**) Bacterial CFU enumerated from the lungs of mice co-inoculated with WT and ∆*purI*. Lines connect CFU from a single mouse (*n* = 15; data from two experiments; *P* by multiple Wilcoxon tests with Bonferroni-Dunn post-test). (**G**) Competitive index of ∆*purI*/WT in the lungs of mice in (**F**). Each dot is from one mouse (median is shown; *P* by one-sample Wilcoxon test compared to 0 and comparison between diets by Mann-Whitney test). (**H**) Bacterial CFU enumerated from the lungs of mice fed standard chow and co-inoculated with WT and either ∆*purI* or ∆*purI* mini-Tn*7*(purI) complement strains. Lines connect CFU from a single mouse (*n* = 9–10; *P* by multiple Wilcoxon tests with Bonferroni-Dunn post-test). (**I**) Competitive index of ∆*purI* strains/WT in the lungs of mice in (**H**). Each dot is from one mouse (median is shown, *P* by one-sample Wilcoxon test compared to 0 and comparison between strains by Mann-Whitney test).

Transcript abundance of *A. baumannii* genes for siderophore biosynthesis and transport and purine biosynthesis had no significant differences between Zn-sufficient and -deficient lungs at 6–24 hpi (Fig. S2A; Fig. 2A). The *barAB* genes encoding the efflux transporter of the siderophore acinetobactin and the *purI* (also known as *purM*) gene encoding phosphoribosylaminoimidazole synthetase for *de novo* purine biosynthesis were chosen for further analysis due to Tn insertions in these genes exhibiting differential selection in the Zn-deficient lung. A ∆*bar* mutant was confirmed to have a defect in growth with iron chelation by ethylenediamine-N,N′-bis((2-hydroxyphenyl)acetic acid) (EDDHA) compared to wild type (Fig. 2B) (7). The ∆*bar* mutant had significantly reduced burdens in the lungs of mice fed either diet (Fig. 2C). In contrast to the Tn-seq results, there was no difference in the competitive index of ∆*bar*/wild type in the lungs of mice fed the ctrl v. low Zn diet (Fig. 2D). Thus, acinetobactin efflux is important for lung infection, regardless of dietary Zn, perhaps due to toxic effects of intracellular siderophore accumulation as reported for *Mycobacterium tuberculosis* (8).

The *∆purI* mutant was confirmed to require exogenous purines, such as adenine, in minimal media, which could be complemented by introducing mini-Tn*7*(*purI*) (Fig. 2E; Fig. S2C and D). Thiamine was included in the growth medium because *purI* mutants are thiamine auxotrophs (9, 10). The ∆*purI* mutant had significantly reduced bacterial burdens in the lungs of mice fed either diet at 24 hpi (Fig. 2F). Consistent with the Tn-seq results, the *∆purI* mutant had a significantly lower competitive index compared to wild type in the lungs of Zn-deficient v. Zn-sufficient mice (Fig. 2G). ATP is a purine molecule that can serve as a host alarm during infection and inflammation (11). We hypothesized that Zn-sufficient diet mice may release more ATP, which *A. baumannii* could degrade and use. Extracellular ATP levels were measured in the previously described bronchial alveolar lavage fluid (BALF) samples from the lungs of mice fed ctrl Zn or low Zn diet (3). However, the higher median BALF ATP in infected mice compared to uninfected mice was not statistically significant, and there was no difference based on diet (Fig. S2E). Finally, ∆*purI* had reduced burdens in mice fed standard chow, which was complemented by mini-Tn*7*(*purI*) (Fig. 2H and I). Together, these data show that *A. baumannii* ∆*purI* has a general defect in lung infection, which is exacerbated in Zn-deficient hosts.

### Conclusions

Dietary Zn deficiency is a major risk factor for lung infections and potentiates *A. baumannii* pneumonia pathogenesis (3). Here, we determined that siderophore transport and purine biosynthesis are important for *A. baumannii* lung infection. However, only disruption of the purine biosynthesis gene *purI* had significantly decreased burdens in the lungs of Zn-deficient mice compared to Zn-sufficient mice. *purI* mutants are auxotrophic for purines and thiamine (vitamin B_1_) due to the shared purine/thiamine biosynthetic pathway (9, 10). However, no thiamine genes were found to be important for lung infection in the Tn-seq. Thus, the defects of the ∆*purI* mutant during infection are likely due to insufficient purines. Purine biosynthesis is required for infection of numerous niches by bacterial pathogens, and the purine regulator controls virulence in *Staphylococcus aureus* ([6, 12–21] and reviewed in [22]). Thus, the host likely limits purines from pathogens, and purine biosynthesis mutants are likely proliferation-deficient during infection. Similar purine limitation in Zn-deficient and -sufficient mice is consistent with our recent report suggesting that Zn deficiency does not significantly alter mouse metabolism (23). The enhanced defect for *∆purI* compared to wild-type *A. baumannii* in mice fed the low Zn diet may be due to increased bacterial proliferation in Zn-deficient animals (Fig. 1D), leading to an increased relative defect for a proliferation-deficient ∆*purI* mutant. These results show that siderophore transport and purine biosynthesis are required for *A. baumannii* lung infection and that host dietary Zn deficiency enhances the competitive advantage of purine biosynthesis. Together, these findings suggest that inhibiting purine biosynthesis in *A. baumannii* and other pathogens may prevent proliferation in the host.

## SUPPLEMENTAL MATERIAL

Supplemental material msphere.00735-25-s0001.pdf Fig. S1 and S2, supplemental materials and methods, and Tables S3 to S5.

Table S1 msphere.00735-25-s0002.xlsx Results of Tn-seq TRANSIT analysis.

Table S2 msphere.00735-25-s0003.xlsx Gene lists used to generate the Venn diagram in Fig. S1.

ASM does not own the copyrights to Supplemental Material that may be linked to, or accessed through, an article. The authors have granted ASM a non-exclusive, world-wide license to publish the Supplemental Material files. Please contact the corresponding author directly for reuse.
