# Supplementary material for: A requirement for Acinetobacter baumannii purine biosynthesis during lung infection is exacerbated by host zinc deficiency
Source: mSphere. 2026 Feb 9;11(3):e00735-25. doi: 10.1128/msphere.00735-25 (PMC13037415; doi:10.1128/msphere.00735-25)
Supplement: Supplemental material — Fig. S1 and S2, supplemental materials and methods, and Tables S3 to S5. [file msphere.00735-25-s0001.pdf]

SUPPLEMENTARY INFORMATION

A requirement for *Acinetobacter baumannii* purine biosynthesis during lung infection is exacerbated by host zinc deficiency

Lauren D. Palmer[a,#], Hannah R. Noel[a], Kacie A. Traina[b], John H. Geary[a,$], Eric P. Skaar[b,#]

[a]Department of Microbiology and Immunology, University of Illinois Chicago, Chicago, IL 60612, USA
[b]Department of Pathology, Microbiology, and Immunology, Vanderbilt University Medical Center, Nashville, TN 37232, USA; Vanderbilt Institute for Infection, Immunology, and Inflammation, Vanderbilt University Medical Center, Nashville, TN 37232, USA

[#]Correspondence: ldpalmer@uic.edu and eric.skaar@vumc.org
[$]Present address: University of South Florida, Tampa, FL

SUPPLEMENTARY FIGURES

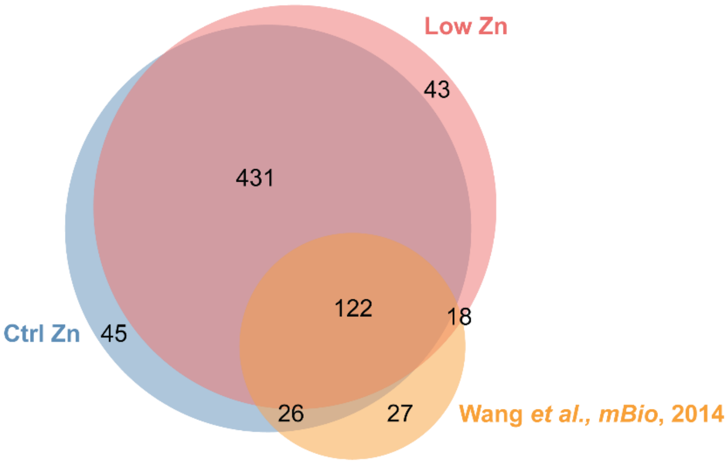

Figure S1: Venn diagram of genes essential for lung infection of mice fed ctrl Zn diet, low Zn diet, or chow as published by Wang *et al*., 2014 (1).
Tn-seq analysis identified genes required for lung infection of the indicated diet group with $|\log_2$ fold-change$| \geq 1$ and adjusted *p* value ≤ 0.05. List of genes represented are shown in supplementary Table S2.

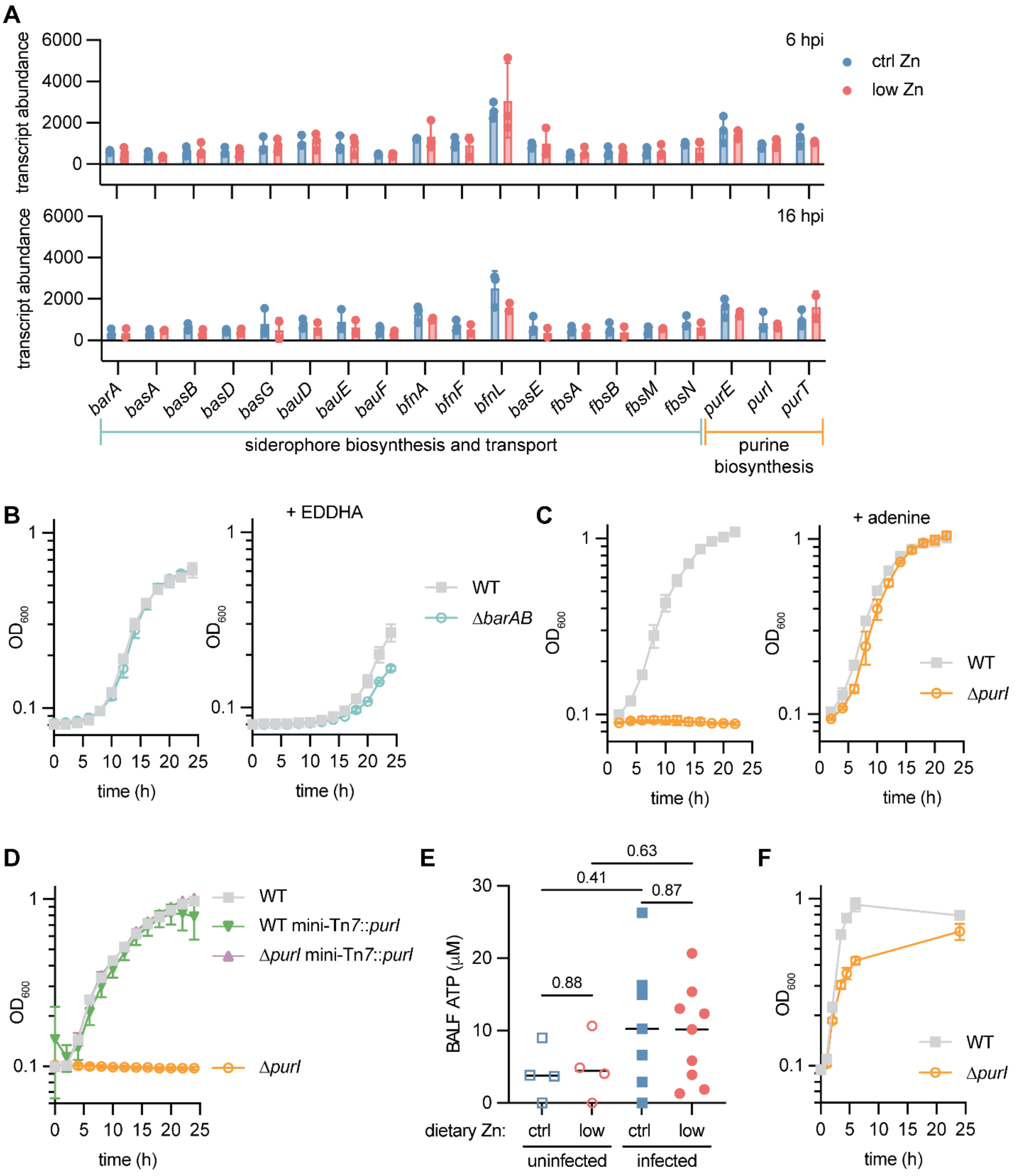

Figure S2: Transcript abundance at earlier timepoints, growth curve data for mutants, and ATP quantification.
(**A**) Transcript abundance in the lungs of mice fed control (ctrl) Zn or low Zn diet at 6 h post infection (hpi) and 16 hpi. Each dot is from one mouse (n = 2-3; mean ± SD; $p \geq 0.4$ by multiple Mann-Whitney tests). (**B**) Growth measured by optical density at 600 nm (OD_{600}) of Δ*barAB* compared to wildtype (WT) in M9 pyruvate metal-free medium with or without iron limitation with ethylenediamine-N,N'-bis((2-hydroxyphenyl)acetic acid) (EDDHA) (n = 2; mean ± SD). (**C**)

Growth measured by OD_{600} of Δ*purI* compared to WT in M9 pyruvate medium with thiamine (10 μM) and with or without adenine (400 μM; n = 4; mean ± SD). (**D**) Growth measured by OD_{600} of WT and Δ*purI* with *purI* complemented from a mini-Tn*7* in M9 pyruvate medium (n = 3; mean ± SD). (**E**) ATP quantified from bronchial alveolar lavage fluid (BALF) samples (2). Each dot is from one mouse (uninfected, n = 4; ctrl Zn infected, n = 7; low Zn infected, n = 9; median is shown; *p* by one-way ANOVA with Holm-Sidak multiple comparisons; data combined from two experiments at 16 hpi and 24 hpi). (**F**) Growth measured by OD_{600} of 0.1 mL aliquots from 10-mL cultures of WT and Δ*purI* inoculated 1:100 in LB similarly to inoculum preparation for infection (n = 3; mean ± SD).

SUPPLEMENTARY MATERIALS AND METHODS

Bacterial strains, plasmids, and growth conditions

Strains and plasmids used in this study are listed in Table S3 and Table S4. Strains were grown in Miller lysogeny broth (LB) or on LB plates including 1.5% w/v agar. Antibiotics were included at the following concentrations: carbenicillin, 75 mg/L; kanamycin, 40 mg/L (20 mg/mL for Δ*bar* mutants); chloramphenicol, 15 mg/L; sulfamethoxazole, 100 mg/L. Each overnight culture was initiated from a single colony and incubated at 37°C with shaking for 8-16 h. To construct deletion strains, plasmid pFLP2 derivatives were constructed from primers in Table S5; *Acinetobacter baumannii* 17978UN Δ*barAB*::Kan was constructed by allelic exchange as previously described (3, 4). *Acinetobacter baumannii* 17978UN Δ*barA*::Kan and Δ*purI*::Kan were constructed by digesting pFLP2-derived vectors digested with BamHI and KpnI, gel purifying the homology-insertion/deletion fragment, and electroporating into a strain containing the pAT02 *A. baumannii* recombinase, similarly to as previously described (5). To complement Δ*purI*::Kan, pKNOCK was digested with BamHI/KpnI and the *purI* promoter and gene were amplified using primers in Table S5, ligated using HiFi (NEB), and transformed into *Escherichia coli* DH5α λ*pir116*. The mini-Tn*7*(*purI*) from sequence-confirmed pKNOCK vectors was introduced into *A. baumannii* 17978UN wildtype (WT) and Δ*purI*::Kn by four-way mating with the *E. coli* HB101 pRK2013 helper strain and *E. coli* pTNS2 Tn*7* transposition plasmid as previously described (3, 4, 6–8).

Animal experiments

Animal experiments were performed similarly to previously described (2). Mouse experiments used C57BL/6J mice (Jackson Laboratories stock no. 000644). For complementation experiments in Figure 2H-I, equal numbers of male and female mice were delivered at 7 weeks of age, maintained on standard chow, and infected at 9 weeks of age. All other mouse experiments with defined diets used male mice delivered at 3-5 weeks of age. We previously reported similar Zn deficiency phenotypes for male and female mice (2, 9). Animals were maintained at the Vanderbilt University Medical Center (VUMC) Animal Facilities or the University of Illinois Chicago (UIC) Biologic Resources Laboratory and food and water were provided *ad libitum*. Animal experiments were performed at VUMC with the exception of one experiment in Figure 2F-G that was performed at UIC. At VUMC, standard chow was LabDiet PicoLab Laboratory Rodent Diet 5L0D. At UIC, standard chow was Teklad LM-485 (catalog #7912). Animals were provided with a 12 h light-dark cycle at VUMC or 14 h light and 10 h dark cycle at UIC. Specialized diets were ordered from Dyets Inc and were L-amino acid defined Zn- and Mn-free AIN-93M diet with Mn added at 10 ppm; low Zn diet had no additional Zn (catalog #515258) and control (ctrl) Zn diet had Zn added to 29 ppm (catalog # 515260). Specialized diets were provided *ad libitum*. Metal analysis by inductively coupled plasma mass spectrometry (ICP-MS) or lab analysis by Dyets Inc. showed that the low Zn diet had <1 ppm Zn (2). Animals were randomized to specialized diets after 3-7 days of acclimation at the facility and groups were ensured to include equivalent initial animal weights.

For mouse infections, *A. baumannii* cultures were grown overnight in 3 mL lysogeny broth (LB) with shaking at 180 rpm at 37°C. The overnight cultures were then diluted 1:100 or 1:1000 in 10 mL LB and grown to mid-logarithmic growth phase for 3.5 h. To reach sufficient density, the Δ*purI* mutant was inoculated 1:20 and grown for 3.5 h or grown for 6 h (*e.g.* Figure S2F). Bacteria were then centrifuged, washed twice in PBS, and resuspended in PBS to ~$1X10^{10}$ colony forming units (CFU)/mL. Mice were anesthetized with intraperitoneal injection of 2,2,2 tribromoethanol or ketamine/xylazine diluted in PBS. *A. baumannii* was then inoculated intranasally with approximately $3X10^{8}$ CFU in 30 µL or mice were mock infected with PBS. Mice were euthanized and organs were removed under sterile conditions and placed on ice. Organs

were homogenized in 1 mL PBS in sterile Whirl-pak bags (Nasco) with a rolling pin or in 0.7 mL PBS using a NextAdvance Bullet Blender tissue homogenizer. Tissue homogenates were serially diluted in a 96-well plate and 5-10 µL samples were plated to LB agar for bacterial CFU enumeration. For competitive infection, mice were inoculated 1:1 with WT and kanamycin-marked deletion strains and CFU were enumerated on LB agar (total *A. baumannii)*, LB with kanamycin, and WT CFU were determined by subtraction. For the Δ*bar* infection, data were combined from infection with WT and Δ*barA* or Δ*barAB*; results were similar from either strain alone. Competitive index was calculated using the following equation: (mutant output/mutant input)/(WT output/WT input).

For experimental endpoints, animals were humanely euthanized in accordance with American Veterinary Medical Association (AVMA) guidelines. All experiments were approved and performed in compliance with the Institutional Animal Care and Use Committee (IACUC) of VUMC (protocol number M1900043-00) and UIC (protocol number 23-119) and conform to policies and guidelines established by VUMC or UIC, the Animal Welfare Act, the National Institutes of Health, and the AVMA.

Tn-seq experiment

A transposon (Tn) library was generated with plasmid pJNW684 introduced to *A. baumannii* 17978UN by electroporation and Tn mutants were selected on LB Kan plates. Two independent libraries were generated, with 25.4k and 9.7k independent colonies harvested in each for a library of 35.1k independent colonies. The library was further incubated in LB Kan with shaking at 37°C for 3 h prior to freezing at -80°C with 10% glycerol. An aliquot of frozen Tn library stock was inoculated in two 10-mL LB cultures and grown with shaking at 37°C for 1.25 h. The bacteria were washed in PBS as described above to generate the input Tn library. Mice fed ctrl Zn or low Zn diet were inoculated with $6X10^8$ CFU Tn library in 30 µL as described above. At 24 hours post infection (hpi), lungs were sterilely excised and homogenized in 1 mL PBS in Whirlpak bags (Nasco). After a portion was removed for enumeration, lung homogenate was diluted 1:10 in LB and incubated at 37°C with shaking for 6 h. Bacterial suspensions were separated from lung tissue, pelleted, and frozen at -20°C until gDNA extraction with the DNeasy Blood & Tissue kit (Qiagen). DNA was digested with MmeI, ligated with Tn-seq adapters, and linearly amplified with biotinylated primer as previously described (10, 11). Adapter and primer sequences are listed in Table S5. PCR products were gel purified, normalized, and sequenced at Tufts University Core Facility Genomics.

Tn-seq analysis

Cutadapt 3.4 was used with the command line options -a ACAGGTTG and --minimum-length 16 to remove adapter sequences, remove low-quality bases, and enforce a minimum length of 16 bp after trimming. Quality was assessed by FastQC 0.11.9. Using bowtie 1.3.0 with the options m 1 --no-unal --best -v 1, the preprocessed reads were aligned to a custom reference file, constructed by concatenating the *Acinetobacter baumannii* ATCC 17978UN chromosome (CP079931.1), with plasmids pAB1 (CP079933.1), pAB2 (CP079934.1), and pAB3 (CP079932.1). The options ensured that no multi-mapping reads were reported and that only reads with one or zero mismatches were counted as alignments. Alignment rates ranged from 23% to 53%. A custom script in python 3.9.12 used the bam alignment files to produce TA insertion site counts and output wig files by sample and contig combinations. TA sites were identified as the last two positions on the 3' end of the trimmed reads; 88% to 99% of reads had a 3' TA site. Genes with differential fitness were identified using the Zero-Inflated Negative Binomial method (ZINB) in TRANSIT 3.2.3 (12, 13). Samples from mice fed ctrl Zn and low Zn diet were compared to determine a $\log_2$ fold change and an adjusted *p* value; adjusted *p* value reported by TRANSIT as 0 was set as 1×10^{-7} for the volcano plot. Venn diagram was

generated with DeepVenn (14). Pathway analysis of differentially selected genes was performed in KEGG Mapper with additional manual curation (15).

Nanostring transcript abundance

Nanostring data were from previously reported samples and additional samples at 6 hpi and 16 hpi using *A. baumannii gyrA* for normalization in nSolver Analysis Software 3.0 (Nanostring) (2). Briefly, right lungs were excised at the indicated time points, flash frozen in liquid nitrogen, and stored at -80°C until RNA extraction. To extract RNA, lung tissue was thawed in Navy Eppendorf RNA Lysis tubes (Next Advance) with Buffer RLT (Qiagen) and homogenized with a Bullet Blender tissue homogenizer (Next Advance) at 4°C. RNA was extracted with the RNeasy mini kit (Qiagen) and transcripts were quantified with a custom NanoString Reporter CodeSet as previously described (16).

Bacterial growth curves

Growth curves contained 100 µL media/well in flat bottom 96-well plates and were inoculated with 1 µL overnight culture and incubated at 37°C with shaking. For growth curves with Δ*barAB* and EDDHA, overnight cultures were first diluted 1/100 in PBS before inoculating media with 1 µL diluted culture. Minimal media were M9 without glucose with 50 mM pyruvate and supplemented with 0.1X Vishniac's trace minerals (17, 18), and thiamine at 100 nM when indicated. For experiments including EDDHA, the trace minerals mix was omitted. Growth was monitored by optical density at 600 nm (OD_{600}) in a BioTek plate reader.

ATP quantification in bronchial alveolar lavage fluid (BALF)

BALF samples were previously described for serum albumin quantification (2). ATP was quantified using the ATPLite luminescence assay kit (Revvity) according to manufacturer's instructions.

Data reporting, statistical analysis, and figure preparation

Each measurement was taken from a distinct biological sample (*e.g.* an individual mouse or bacterial culture from a single colony). Data processing and statistical analyses were performed using Microsoft Excel 16.77.1 and GraphPad Prism 10.5.0 unless otherwise specified. Values below the limit of detection are graphed at the limit of detection for statistical purposes. Statistical tests are listed in figure legends. All *p* values for *t* tests and Mann-Whitney tests are two-tailed. Transcript abundance normalization to *gyrA* was calculated with nSolver Analysis Software 3.0 (Nanostring). Figures were prepared in Adobe Illustrator 28.7.8.

Data and code availability

Tn-seq sequencing data are publicly available in the National Center for Biotechnology Information Sequence Read Archive (SRA) BioProject PRJNA1321337 (19). Code for Tn-seq analysis is available at https://github.com/trestle-biosciences/palmer_lab_a_baumannii_zn_diet_tnseq.

Table S1. Results of Tn-seq TRANSIT analysis.

Table S2. Gene lists used to generate Venn diagram in Figure S1.

Table S3. Strains.

Name	Source	Number
Acinetobacter baumannii 17978UN	Wijers *et al.*, 2021 (20)	LP303
Acinetobacter baumannii 17978UN pAT02	This study	LP147
Acinetobacter baumannii 17978UN Δ*barA*::Kan	This study	LP265
Acinetobacter baumannii 17978UN Δ*barAB*::Kan	This study	LP281
Acinetobacter baumannii 17978UN Δ*purI*::Kan	This study	LP266
Acinetobacter baumannii 17978UN *att*::mini-Tn*7*(*purI*)	This study	LP1341
Acinetobacter baumannii 17978UN Δ*purI*::Kan *att*::mini-Tn*7*(*purI*)	This study	LP1342

Table S4. Plasmids.

Number	Name	Description	Source
pJNW684		Transposon vector containing Kan^R *himar1* transposon. Carb^R	Wang *et al.*, 2014 (1)
pFLP2		Allelic exchange vector. Carb^R Sucrose^S	Hoang *et al.*, 1998 (21)
pUC18-K1		Non-polar kanamycin resistance cassette. Kan^R	Ménard et al., 1993 (22)
pRK2013		Mobilization helper plasmid. Kan^R	Figurski and Helinski, 1979 (8)
pLDP45	pFLP2-*barA*::Kan	Allelic exchange vector to generate Δ*barA*::Kan. Carb^R Kan^R Sucrose^S	This study
pLDP47	pFLP2-*purI*::Kan	Allelic exchange vector to generate Δ*purI*::Kan. Carb^R Kan^R Sucrose^S	This study
pLDP51	pFLP2-*barAB*::Kan	Allelic exchange vector to generate Δ*purI*::Kan. Carb^R Kan^R Sucrose^S	This study
pAT02		pREC-ET-carb	Tucker *et al.*, 2014 (5)
pTNS2		Helper plasmid encoding the site-specific TnsABCD Tn7 transposition pathway. Carb^R	Kumar *et al.*, 2010 (7)
pKNOCK		Mini-Tn*7*-Carb^R on a suicide vector containing the R6K γ-ori	Carruthers *et al.*, 2013 (6)
pLDP387	pKNOCK-*purI*	Mini-Tn*7*-Carb^R encoding *purI* with endogenous promoter	This study

Table S5. Oligonucleotides.

All oligonucleotides were purchased from Integrated DNA Technologies (IDT).

Name	Sequence
ADBC-F-INDX1a	ACACGACGCTCTTCCGATCTGGAACTCCTACTGACNN
ADBC-F-INDX1b	ACACGACGCTCTTCCGATCTTAGCACATGCTGACTNN
ADBC-F-INDX1c	ACACGACGCTCTTCCGATCTCCTTGAGGATGACTGNN
ADBC-F-INDX1d	ACACGACGCTCTTCCGATCTATCGTGTACGACTGANN
ADBC-F-INDX2a	ACACGACGCTCTTCCGATCTGACTACCCTCTGGACNN
ADBC-F-INDX2b	ACACGACGCTCTTCCGATCTTCTGGATACACTACTNN

ADBC-F-INDX2c	ACACGACGCTCTTCCGATCTCTGATGGGAGACCTGNN
ADBC-F-INDX2d	ACACGACGCTCTTCCGATCTAGACCTATGTGATGANN
ADBC-F-INDX3a	ACACGACGCTCTTCCGATCTGACTGCCACATGAACNN
ADBC-F-INDX3b	ACACGACGCTCTTCCGATCTTCTGAATCTCCTGCTNN
ADBC-F-INDX3c	ACACGACGCTCTTCCGATCTAGAACGGTGTACTTGNN
ADBC-F-INDX3d	ACACGACGCTCTTCCGATCTCTGCTTAGAGGACGANN
ADBC-F-INDX4a	ACACGACGCTCTTCCGATCTCGACACTGAACGACCNN
ADBC-F-INDX4b	ACACGACGCTCTTCCGATCTTTCTGACTCCTTGATNN
ADBC-F-INDX4c	ACACGACGCTCTTCCGATCTGCTGTGACTTGCTGGNN
ADBC-F-INDX4d	ACACGACGCTCTTCCGATCTAAGACTGAGGAACTANN
ADBC-F-INDX6a	ACACGACGCTCTTCCGATCTCCTAAAGGAATGGCCNN
ADBC-F-INDX6b	ACACGACGCTCTTCCGATCTATGGCGTACCCTAATNN
ADBC-F-INDX6c	ACACGACGCTCTTCCGATCTGGATTTCCTTACCGGNN
ADBC-F-INDX6d	ACACGACGCTCTTCCGATCTTACCGCATGGGATTANN
ADBC-R-INDX1a	/5PHOS/GTCAGTAGGAGTTCCAGATCGGAAGAGCGTCGTGTAGGGA/3PHOS/
ADBC-R-INDX1b	/5PHOS/AGTCAGCATGTGCTAAGATCGGAAGAGCGTCGTGTAGGGA/3PHOS/
ADBC-R-INDX1c	/5PHOS/CAGTCATCCTCAAGGAGATCGGAAGAGCGTCGTGTAGGGA/3PHOS/
ADBC-R-INDX1d	/5PHOS/TCAGTCGTACACGATAGATCGGAAGAGCGTCGTGTAGGGA/3PHOS/
ADBC-R-INDX2a	/5PHOS/GTCCAGAGGGTAGTCAGATCGGAAGAGCGTCGTGTAGGGA/3PHOS/
ADBC-R-INDX2b	/5PHOS/AGTAGTGTATCCAGAAGATCGGAAGAGCGTCGTGTAGGGA/3PHOS/
ADBC-R-INDX2c	/5PHOS/CAGGTCTCCCATCAGAGATCGGAAGAGCGTCGTGTAGGGA/3PHOS/
ADBC-R-INDX2d	/5PHOS/TCATCACATAGGTCTAGATCGGAAGAGCGTCGTGTAGGGA/3PHOS/
ADBC-R-INDX3a	/5PHOS/GTTCATGTGGCAGTCAGATCGGAAGAGCGTCGTGTAGGGA/3PHOS/
ADBC-R-INDX3b	/5PHOS/AGCAGGAGATTCAGAAGATCGGAAGAGCGTCGTGTAGGGA/3PHOS/
ADBC-R-INDX3c	/5PHOS/CAAGTACACCGTTCTAGATCGGAAGAGCGTCGTGTAGGGA/3PHOS/
ADBC-R-INDX3d	/5PHOS/TCGTCCTCTAAGCAGAGATCGGAAGAGCGTCGTGTAGGGA/3PHOS/
ADBC-R-INDX4a	/5PHOS/GGTCGTTCAGTGTCGAGATCGGAAGAGCGTCGTGTAGGGA/3PHOS/
ADBC-R-INDX4b	/5PHOS/ATCAAGGAGTCAGAAAGATCGGAAGAGCGTCGTGTAGGGA/3PHOS/
ADBC-R-INDX4c	/5PHOS/CCAGCAAGTCACAGCAGATCGGAAGAGCGTCGTGTAGGGA/3PHOS/
ADBC-R-INDX4d	/5PHOS/TAGTTCCTCAGTCTTAGATCGGAAGAGCGTCGTGTAGGGA/3PHOS/
ADBC-R-INDX6a	/5PHOS/GGCCATTCCTTTAGGAGATCGGAAGAGCGTCGTGTAGGGA/3PHOS/
ADBC-R-INDX6b	/5PHOS/ATTAGGGTACGCCATAGATCGGAAGAGCGTCGTGTAGGGA/3PHOS/
ADBC-R-INDX6c	/5PHOS/CCGGTAAGGAAATCCAGATCGGAAGAGCGTCGTGTAGGGA/3PHOS/
ADBC-R-INDX6d	/5PHOS/TAATCCCATGCGGTAAGATCGGAAGAGCGTCGTGTAGGGA/3PHOS/
BioSamA	/BIO/TEG/CAAGCAGAAGACGGCATACGAAGACC
ADPT-Tnseq-PCRPrimer	AATGATACGGCGACCACCGAGATCTACACTCTTTCCCTACACGACGCTCTTCCGATCT

Genomic DNA sequencing primer	ACACTCTTTCCCTACACGACGCTCTTCCGATCT
pUC18-K1 kanamycin resistance cassette F	cccgggTGACTAACTAGGAGGA
pUC18-K1 kanamycin resistance cassette R	cccgggTCATTATTCCCTCCAG
pFLP2-barA-up-F	gttaaaaaggatcgatcctctagaggatccTTGAAATGATTCAAGAGCGC
pFLP2-barA-up-R	ttattcctcctagttagtcagccccacagagcagttaaaatg
pFLP2-barA-down-F	gtacctggagggaataatgaATACTGCTGCTAAATTAAATAAAGATC
pFLP2-barA-down-R	atgattacgaattcgagctcggtaccGGCGCATAGCTAATGC
barA_up_up_F	ACCGGCGCAATTGATG
barA_dn_dn_R	CTCCCCAATGCGATATCG
purIup_pFLP2_F	aaaggatcgatcctctagaggatcCTGCCTGCCCAATCATAAAG
purIup_aphA_R	ttattcctcctagttagtcaAAATGAAGTCTCCGCATTG
purIdn_aphA_F	gtacctggagggaataatgaATGATGAAAATTGCTG
purIdn_pFLP2_R	tacgaattcgagctcggtaccTGACTATGCTTTGAAAG
purI_up_up_F	GTAGGAATGGCTGCAG
purI_dn_dn_R	GCTGCATTGTTACGCC
barBdn_aphA_F	ttgttttagtacctggagggaataatgaAAAAATAGAGCAGAAATCTGATTTAAG
barBdn_pFLP2_R	atgattacgaattcgagctcggtaccTATATTCGGTGGAAGAAATATATTC
barB_dn_dn_R	ACGAATGAAGCGTGAG
pKNOCK-purI-fwd	tcatgcatgagctcactagtggatcaaaatctagcctttttgatttaat
pKNOCK-purI-rev	ggcctgcaaggccttcgcgaggtactcattcaaagatcacacg

SUPPLEMENTARY REFERENCES

1. Wang N, Ozer EA, Mandel MJ, Hauser AR. 2014. Genome-wide identification of *Acinetobacter baumannii* genes necessary for persistence in the lung. mBio 5:e01163-14.

2. Palmer LD, Traina KA, Juttukonda LJ, Lonergan ZR, Bansah DA, Ren X, Geary JH, Pinelli C, Boyd KL, Yang TS, Skaar EP. 2024. Dietary zinc deficiency promotes *Acinetobacter baumannii* lung infection via IL-13 in mice. Nat Microbiol 9:3196–3209.

3. Palmer LD, Minor KE, Mettlach JA, Rivera ES, Boyd KL, Caprioli RM, Spraggins JM, Dalebroux ZD, Skaar EP. 2020. Modulating isoprenoid biosynthesis increases lipooligosaccharides and restores *Acinetobacter baumannii* resistance to host and antibiotic stress. Cell Rep 32:108129.

4. Noel HR, Keerthi S, Ren X, Winkelman JD, Troutman JR, Palmer LD. 2023. Genetic synergy in *Acinetobacter baumannii* undecaprenyl biosynthesis and maintenance of lipid asymmetry impacts outer membrane and antimicrobial resistance. 556980 https://doi.org/10.1101/2023.09.22.556980.

5. Tucker AT, Nowicki EM, Boll JM, Knauf GA, Burdis NC, Trent MS, Davies BW. 2014. Defining gene-phenotype relationships in *Acinetobacter baumannii* through one-step chromosomal gene inactivation. MBio 5:e01313-14.

6. Carruthers MD, Nicholson PA, Tracy EN, Munson RS. 2013. *Acinetobacter baumannii* utilizes a type VI secretion system for bacterial competition. PLoS ONE 8:e59388.

7. Kumar A, Dalton C, Cortez-Cordova J, Schweizer HP. 2010. Mini-Tn*7* vectors as genetic tools for single copy gene cloning in *Acinetobacter baumannii*. J Microbiol Methods 82:296–300.

8. Figurski DH, Helinski DR. 1979. Replication of an origin-containing derivative of plasmid RK2 dependent on a plasmid function provided *in trans*. Proc Natl Acad Sci U A 76:1648–52.

9. Murdoch CC, Weiss A, Enriquez KT, Traina KA, Drury SL, Winn NC, Lantier LL, Skaar EP. 2025. Severe dietary zinc deficiency does not significantly alter energy balance in adult mice. J Nutr Metab 2025:6911386.

10. van Opijnen T, Bodi KL, Camilli A. 2009. Tn-seq: high-throughput parallel sequencing for fitness and genetic interaction studies in microorganisms. Nat Methods 6:767–772.

11. Goodman AL, Wu M, Gordon JI. 2011. Identifying microbial fitness determinants by insertion sequencing using genome-wide transposon mutant libraries. Nat Protoc 6:1969–1980.

12. DeJesus MA, Ambadipudi C, Baker R, Sassetti C, Ioerger TR. 2015. TRANSIT - a software gool for *himar1* TnSeq analysis. PLOS Comput Biol 11:e1004401.

13. Subramaniyam S, DeJesus MA, Zaveri A, Smith CM, Baker RE, Ehrt S, Schnappinger D, Sassetti CM, Ioerger TR. 2019. Statistical analysis of variability in TnSeq data across conditions using zero-inflated negative binomial regression. BMC Bioinformatics 20.

14. Hulsen T. 2022. DeepVenn -- a web application for the creation of area-proportional Venn diagrams using the deep learning framework Tensorflow.js (1). arXiv https://doi.org/10.48550/ARXIV.2210.04597.

15. Kanehisa M, Furumichi M, Sato Y, Matsuura Y, Ishiguro-Watanabe M. 2025. KEGG: biological systems database as a model of the real world. Nucleic Acids Res 53:D672–D677.

16. Sheldon JR, Skaar EP. 2020. *Acinetobacter baumannii* can use multiple siderophores for iron acquisition, but only acinetobactin is required for virulence. PLoS Pathog 16:e1008995.

17. M9 salt solution. Cold Spring Harb Protoc 2009:pdb.rec11973.

18. Vishniac W, Santer M. 1957. The thiobacilli. Bacteriol Rev 21:195–213.

19. Palmer LD, Noel HR, Traina KA, Geary JH, Skaar, EP. 2025. *Acinetobacter baumannii* purine biosynthesis is required for lung infection of zinc deficient mice. PRJNA1321337. Raw sequence reads. Sequence Read Archive. https://www.ncbi.nlm.nih.gov/bioproject/?term=PRJNA1321337.

20. Wijers CDM, Pham L, Menon S, Boyd KL, Noel HR, Skaar EP, Gaddy JA, Palmer LD, Noto MJ. 2021. Identification of two variants of *Acinetobacter baumannii* strain ATCC 17978 with distinct genotypes and phenotypes. Infect Immun 89:e0045421.

21. Hoang TT, Karkhoff-Schweizer RR, Kutchma AJ, Schweizer HP. 1998. A broad-host-range Flp-FRT recombination system for site-specific excision of chromosomally-located DNA sequences: application for isolation of unmarked Pseudomonas aeruginosa mutants. Gene 212:77–86.

22. Ménard R, Sansonetti PJ, Parsot C. 1993. Nonpolar mutagenesis of the *ipa* genes defines IpaB, IpaC, and IpaD as effectors of *Shigella flexneri* entry into epithelial cells. J Bacteriol 175:5899–5906.
